# Supplementary material for: Served Portion Sizes Affect Later Food Intake Through Social Consumption Norms
Source: Nutrients. 2019 Nov 20;11(12):2845. doi: 10.3390/nu11122845 (PMC6949930; doi:10.3390/nu11122845)
Supplement: Supplementary file 1 [file nutrients-11-02845-s001.pdf]

Supplementary Materials

Methods

Study 1

Power analysis

The sample size was determined based on a Monte Carlo power analysis for indirect effects using an online application [18]. Power estimations indicate that in a model with two parallel mediators, a power of .80 with an alpha level of $p < .05$ is reached with 235 participants, assuming correlations of $r = 0.3$ between the independent variable X, the dependent variable Y and the mediators M. However, four parallel mediators were included in the analysis (three different norm measures and the satiety measure) and we planned to exclude participants who guessed the study aims, hence the sample size was increased to 330 participants (approximately 110 participants/condition).

Pilot study: Portion size stimuli selection

An online pilot study conducted with 20 University of Liverpool employees (65% female, M age = 28.65 years, SD = 6.29) was used to select portion size stimuli for the exposure manipulation. Participants viewed standardized images of 27 different portion sizes of each food ranging from 40% to 300% of a reference portion in 10% increments in an online questionnaire programmed in Qualtrics. The reference portion was equal to the manufacturer's recommended serving (300g cooked lasagna (569kcal) and 210g cooked spaghetti (506kcal)). Whether participants first evaluated lasagna or spaghetti was evenly randomized across participants, as was the portion size presentation sequence within each food type. For each portion size, participants indicated whether they perceived it to be a 'normal' or 'not normal' amount to eat (order of response options was evenly randomized). A relatively small portion (60% of reference portion: 180g cooked lasagna, 341 kcal) and a relatively large portion (180% of reference portion: 540g cooked lasagna, 1024 kcal) of lasagna that were perceived to be beyond the boundaries of a normal portion by the majority of participants were selected as stimuli for the initial exposure phase (see Figure 1 (main manuscript file), and see Supplementary Figure S1 and S2 for the pilot study results). Portion sizes that were outside of the range perceived as 'normal' were selected because this study aims to examine whether portion size norms adjust to these initially perceived 'not normal' smaller (versus larger) portion sizes as one could argue that the range of portion sizes initially classed as being 'normal' in size has been shifted upwards in recent decades.

Hunger and liking

Hunger was measured with one item [10]: 'think back to just before you were about to start the questionnaire today. How hungry were you?', with responses reported on a 9-point Likert scale ranging from 1 (not at all hungry) to 9 (extremely hungry).

Liking was measured with one item for the two foods separately: 'how much do you like (lasagna/spaghetti)?', with responses collected on 9-point Likert scales ranging from 1 (not at all) to 9 (very much).

Funneled manipulation check

First, participants were asked 'what image did you view in yesterday's questionnaire?', to which they responded by selecting one image from six images (three images of non-food objects: the printer image plus two filler images, and three food images: an image of lasagna plus two filler images). Participants who selected the image of lasagna in the first question were asked 'what portion size did you view in yesterday's questionnaire?', to which they responded by selecting one of the nine portion size images (as in Robinson and Kersbergen [7]). Participants who correctly selected the 'printer' image in response to the first question, and participants in either portion size condition who both (a) selected the 'lasagna' image in the first question and (b) selected a portion that was either the same size or one

size smaller or larger than the portion that they viewed in the first session, were coded as correctly recalling the manipulation.

Exclusion criteria for analysis

Awareness of the aims of the study was measured with the following open-ended question: 'what do you think the aims of the study were?' Responses were independently coded by two researchers as being aware or unaware of the aims of the study (100% agreement between researchers). Participants who linked the portion size they were exposed to in session one to portion size selection or normality measures during session two were categorized as having identified the aim of the study.

Food allergies or intolerances were assessed with the open-ended question: 'do you have any allergies, intolerances or specific dietary requirements (e.g. vegetarian)?'

Study 2

Power analysis

The sample size was determined based on a Monte Carlo power analysis for indirect effects using an online application [18]. Power estimations indicate that in a model with two parallel mediators, a power of 0.80 ($p < 0.05$) is reached with 99 participants. Based on the correlations observed in Study 1 and in previous research [7], correlations of $r = 0.4$ were assumed between the independent variable X, and the mediators M, and the dependent variable Y. Correlations of $r = 0.5$ were assumed between the mediators M and the dependent variable Y, as well as between the mediators themselves. However, four parallel mediators were included in the analysis and we planned to exclude participants who guessed the study aims, hence the sample size was increased to 140 participants (approximately 70 participants/condition).

Pilot study: Portion size stimuli selection

An online pilot study conducted with Dutch female participants was used to select portion size stimuli for the exposure manipulation, following a similar procedure to the pilot study prior to Study 1. Participants viewed standardized images of 30 different portion sizes ranging from 10% to 300% of the reference portion. The range of portions presented in the pilot study was extended to a minimum of 10% of the reference portion (compared to a minimum of 40% in the pilot study for Study 1) to avoid a floor effect, as in the pilot study for Study 1 participants' perceived 'normal' range of portions was located at the lower end of the scale. In total, 102 participants completed the pilot study, of which 52 participants were included in analyses[1]. The analytic sample comprised of Dutch female participants, including students (83%) and employees of Wageningen University and Research (M age = 23.80 years, SD = 4.12, *n* = 51; M BMI = 21.65, SD = 2.04, *n* = 51). A relatively small portion (60% of reference portion) and a relatively large portion (180% of reference portion) of lasagna that were perceived to be beyond the boundaries of a normal portion by the majority of participants were selected for the manipulation phase (see Supplementary Figure S1 for pilot study results). The beef lasagna served in Study 2 (Figure 2 (main manuscript file)) was similar in appearance to the beef lasagna shown in the pilot study and Study 1, although the nutritional value (kcal) somewhat differed (reference portion (Aldi supermarket): 300g cooked lasagna (486 kcal)).

Filler task: mood questionnaires

To bolster the cover story, participants completed mood questionnaires before and after eating lunch. The 8-item filler mood questionnaire was programmed on Qualtrics and included items about

---

[1] Participants who did not follow instructions by using a mobile phone instead of a laptop or desktop computer (*n* = 17), males (*n* = 1) and participants who indicated they were following a diet or did not consume lasagna because of allergies, intolerances or dietary specific requirements (e.g., vegetarian) (*n* = 32) were excluded from analyses.

participants' current mood (e.g., "how relaxed do you feel right now?"), items measuring appetite, and one item asking participants to report how long since they last ate. Questionnaire items were presented in an evenly randomized order.

Hunger, liking, frequency of consumption, and awareness of monitoring consumption

Hunger was measured with two items that were averaged into a single score [7] (Cronbach's α = 0.89): 'how hungry do you feel right now?' (with responses on a 9-point scale ranging from 1 (not at all hungry) to 9 (extremely hungry)) and 'how full do you feel right now?' (reverse coded, with responses on a 9-point scale ranging from 1 (not at all full) to 9 (extremely full)).

Liking of lasagna was measured with one item (as in Robinson and Kersbergen [7]): 'how much did you like the lasagna?' (with responses on a 9-point scale ranging from 1 (not at all tasty) to 9 (extremely tasty)).

Frequency of eating lasagna was measured with one item (as in Robinson and Kersbergen [7]): 'how often do you eat lasagna?' (with responses on a 9-point scale ranging from 1 (never) to 9 (everyday)).

Participants' awareness that their consumption was being monitored by the researcher was assessed with one item (as in Robinson and Kersbergen [7]): 'did you feel that the amount of lasagna you were eating was measured by the researcher' (with responses on a 9-point scale ranging from 1 (not at all) to 9 (very much)).

Manipulation check

As in Study 1 participants were asked: 'how much of this lasagna were you served yesterday?' and were instructed to select one of the nine lasagna portion images (see Figure 1 [main manuscript file]). Participants who selected a portion that was either the same size or one size smaller or larger than the portion that they consumed in the first session were coded as correctly recalling the manipulation.

Exclusion criteria for analysis

Awareness of the aims of the study was assessed using the same methodology as in Study 1. Agreement for coding decisions between two independent researchers was 100%.

Adherence to abstinence requirements was measured with the following item: 'how long ago did you last eat?' (options: 30 minutes ago, 1 hour ago, 1.5 hour ago, 2 hours ago, 2.5 hours ago, 3 hours ago and longer than 3 hours ago).

Additional planned analysis strategy

As secondary analysis, two separate independent-sample *t*-tests were performed to examine the effect of portion size condition on (1) salad consumption during day 1 and (2) salad consumption during day 2.

As part of the sensitivity analysis, linear regression modelling was included to test whether the effect of portion size condition on consumption during the second session was dependent on awareness of consumption being monitored by the researcher (to explore demand characteristics). Portion size condition was entered in the first step of a forced entry model and in the second step mean centered awareness of participants and mean centered condition * awareness of participants interaction was entered.

Additional Results

Study 1

Sensitivity analysis

The statistical significance of the results did not differ after inclusion of covariates that were significantly correlated with outcome or potential mediator variables (see correlations in Supplementary Table S2), except for the effect of condition on perceptions of injunctive social norms for incongruent foods (spaghetti). An ANCOVA testing this effect including sex, exposure duration and hunger as covariates, showed no significant effect ($F(2, 321) = 0.41$, $p = 0.67$, $\eta p^2 = 0.003$, $n = 327$).

Study 2

Secondary consumption analysis

Participants who were served the smaller (as opposed to the larger) portion size consumed marginally significantly more salad during session one, while salad consumption during session two did not significantly differ between participants in the smaller and larger portion size condition (see Supplementary Table S7).

Sensitivity analysis

The statistical significance of the results did not differ after inclusion of covariates that were significantly correlated with outcome or potential mediator variables (see correlations in Supplementary Table S5), except for the specific mediating role of descriptive social norms in the effect of portion size condition on later consumption. After controlling for age, baseline hunger (session two), liking (session two) and frequency of eating lasagna, mediation analysis still indicated a significant total indirect effect of portion size condition on later consumption via perceptions of descriptive social norms and injunctive social norms (indirect effect = 15.87, SE = 7.40, 95% CI (3.11, 31.88), proportion of total effect explained by indirect effect = 18.93%). However, both specific indirect effects were non-significant (descriptive social norms: indirect effect = 11.44, SE = 7.37, 95% CI (-0.32, 27.85), proportion of total effect explained by indirect effect = 13.64%; injunctive social norms: indirect effect = 4.43, SE = 6.54, 95% CI (-7.36, 19.15), proportion of total effect explained by indirect effect = 5,28%).

To explore the role of demand characteristics, we tested whether the effect of portion size condition on later consumption was dependent on participants' awareness of their consumption being monitored by the researcher. Consistent with primary analyses, portion size condition significantly predicted later consumption ($\beta = 0.37$, $p < 0.001$), and neither consumption the next day nor the effect of condition on next day consumption was impacted by how aware participants were of their consumption being monitored by the researcher (awareness of monitoring consumption: $\beta = 0.17$, $p = 0.52$; interaction: $\beta = -0.04$, $p = 0.87$).

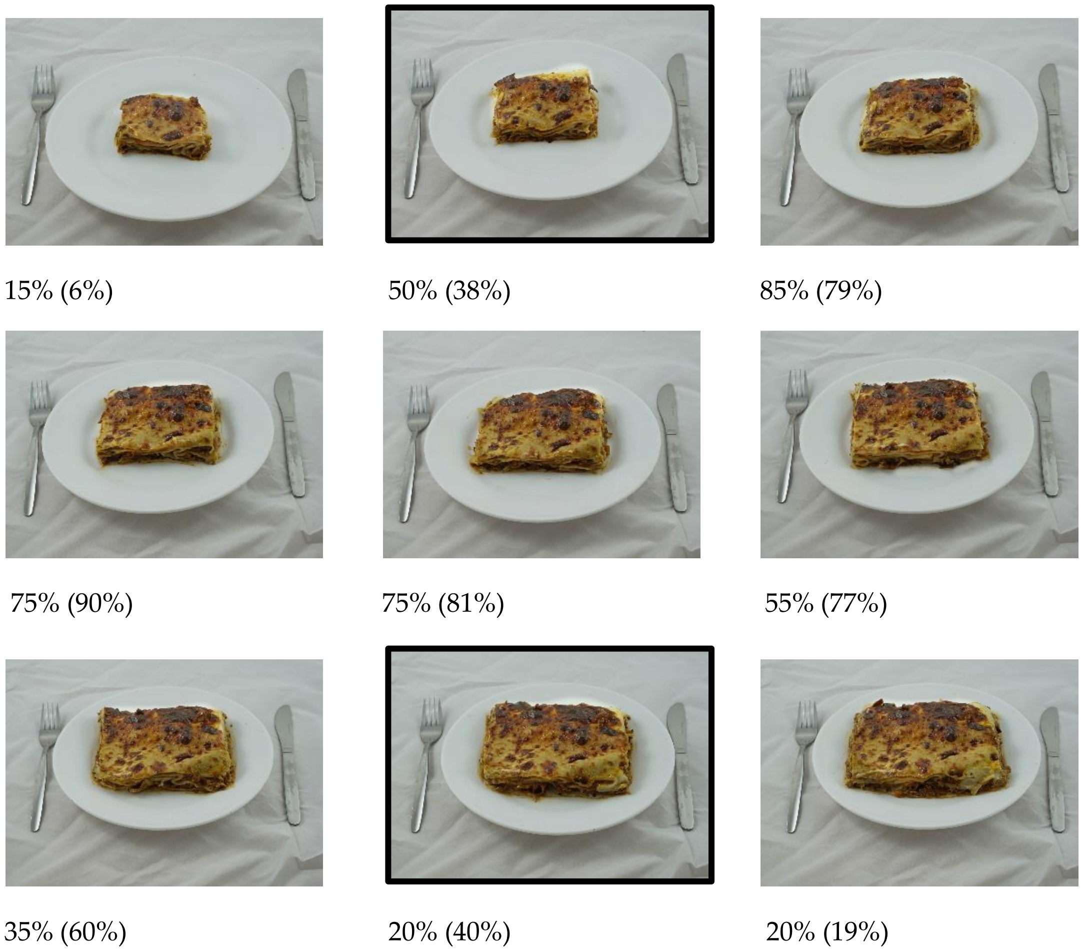

Figure S1. Portion size scale for lasagna based on pilot study results of Study 1 (Study 2). The percentages below the portions reflect the proportion of participants judging the portion as a 'normal' amount to eat. Results of Study 2 are shown between brackets.

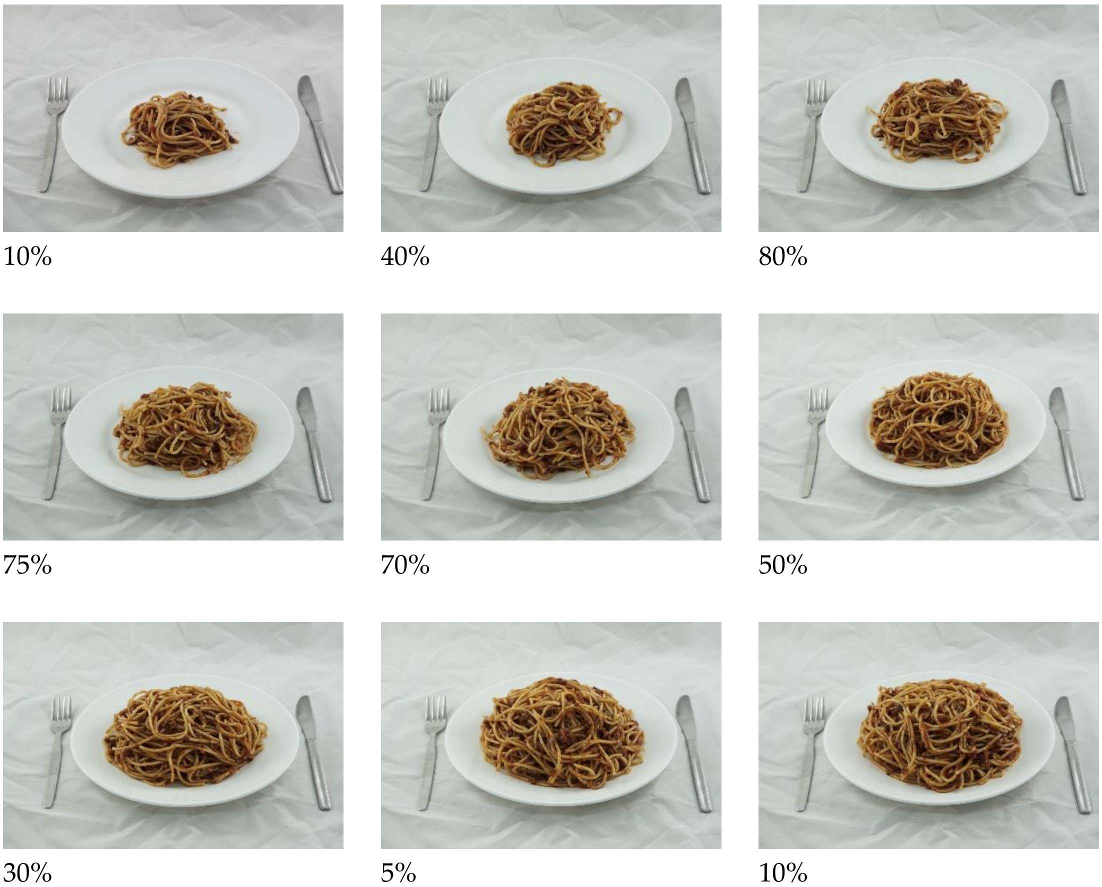

Figure S2. Portion size scale for spaghetti based on pilot study results of Study 1. The percentages below the portions reflect the proportion of participants judging the portion as a 'normal' amount to eat.

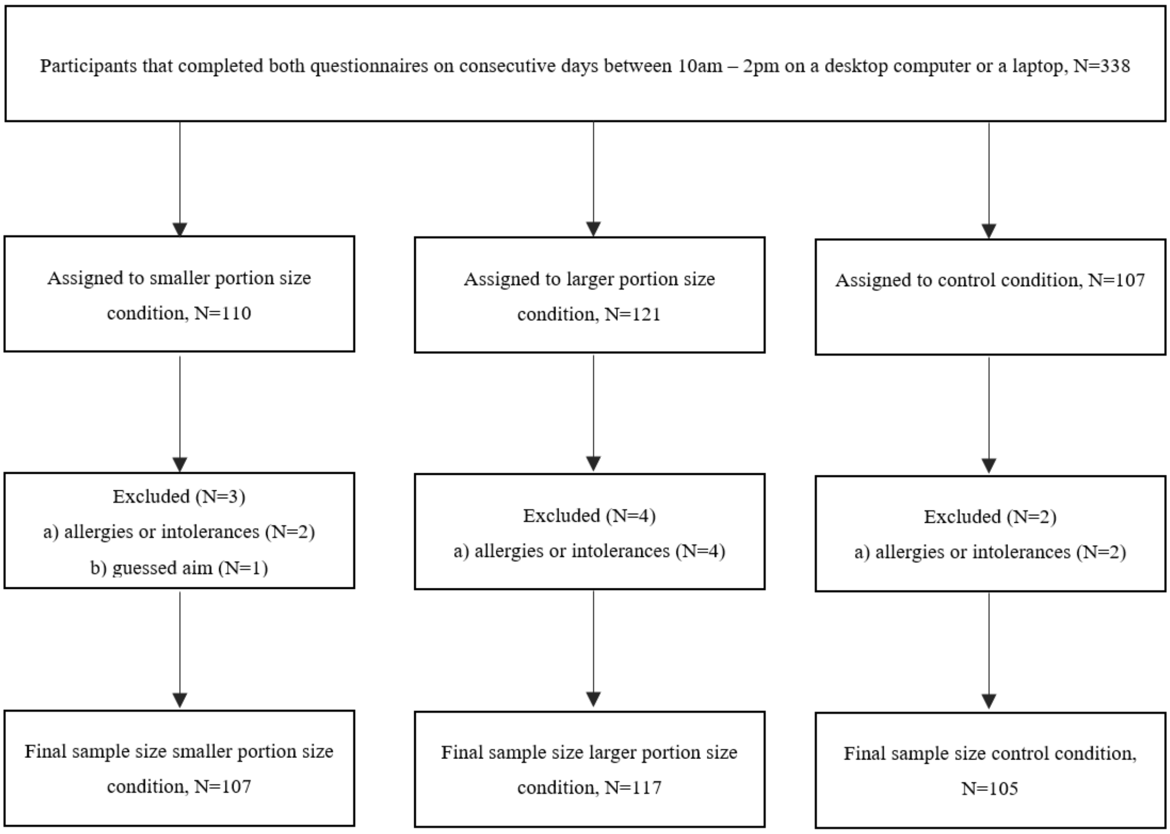

Figure S3. Flowchart of participants (Study 1).

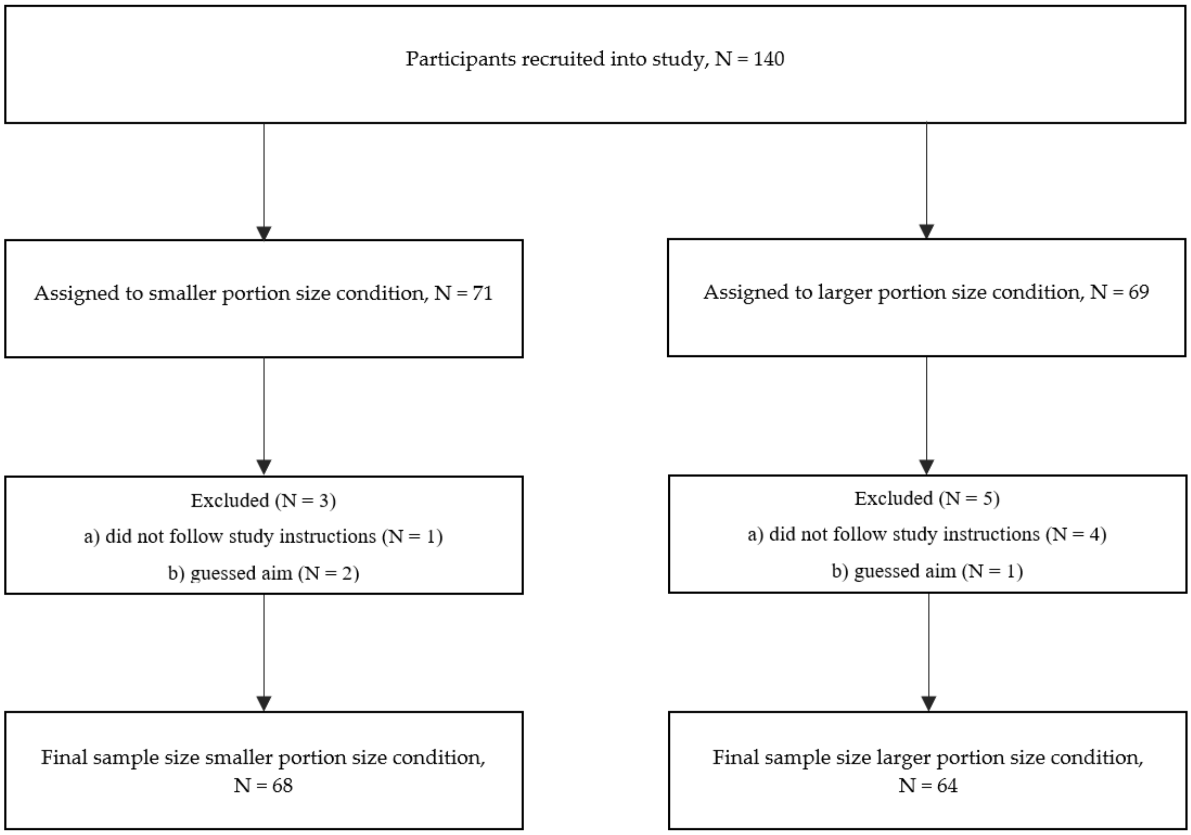

Figure S4. Flowchart of participants (Study 2). Reasons for exclusion because of not following study instructions included (1) not filling in the last questionnaire after consumption during session two (n = 1, smaller portion size condition), (2) not adhering to abstinence requirements (n = 3, larger portion size condition) and (3) only consuming 12 grams of lasagna during session two which indicated either a measurement error or an unusual value (n = 1, larger portion size condition).

Table S1. Component paths of the indirect effect of condition on portion size selection for each proposed mediator (n = 329, Study 1).

		**Relation between condition and proposed mediator**	**Relation between proposed mediator and portion size selection**
		B (95% CI)	*B* (95% CI)
Perceptions of portion size normality	Lasagna	0.02 (-0.04, 0.09)	0.18 (0.15, 0.22)
	Spaghetti	0.01 (-0.04, 0.07)	0.24 (0.20, 0.27)
Perceptions of descriptive social norms	Lasagna	0.03 (-0.05, 0.10)	0.14 (0.10, 0.17)
	Spaghetti	0.05 (-0.02, 0.11)	0.14 (0.11, 0.17)
Perceptions of injunctive social norms	Lasagna	0.03 (-0.04, 0.10)	0.12 (0.09, 0.16)
	Spaghetti	0.06 (-0.001, 0.13)	0.15 (0.11, 0.18)
Personal norms	Lasagna	-0.003 (-0.07, 0.07)	0.24 (0.21, 0.27)
	Spaghetti	0.01 (-0.05, 0.07)	0.26 (0.23, 0.28)
Expected satiety	Lasagna	-0.01 (-0.07, 0.06)	0.23 (0.20, 0.25)
	Spaghetti	-0.01 (-0.07, 0.06)	0.24 (0.21, 0.26)

B = unstandardized regression coefficient. CI = confidence interval.

Table S2. Pearson's correlations between main variables (n = 329 [c], Study 1).

Variable	1	2	3	4	5	6	7	8	9	10	11	12	13	14	15	16	17	18	19	20
1. Age (n = 325)	-																			
2. Sex (female, n = 327) [b]	.02	-																		
3. BMI (kg/m^2, n = 321)	.08	.02	-																	
4. Exposure duration (mm:ss)	.04	-.09	.05	-																
5. Hunger [a]	-.15**	-.06	.09	.06	-															
6. Overall-liking (lasagna) [a]	-.01	.07	.09	.04	.08	-														
7. Overall-liking (spaghetti) [a]	-.02	-.01	.07	.05	.18**	.48**	-													
8. Ethnicity (white) [b]	-.14*	-.03	-.06	.05	.04	-.02	-.11*	-												
9. Portion size selection (lasagna) [a]	-.07	-.21**	.14*	.04	.25**	.31**	.20**	.08	-											
10. Perceptions of portion size normality (lasagna) [a]	.00	-.26**	-.01	.10	.09	.07	-.04	.04	.50**	-										
11. Perceptions of descriptive social norms (lasagna) [a]	.02	-.60**	-.02	.08	.15**	.04	.04	.05	.44**	.60**	-									
12. Perceptions of injunctive social norms (lasagna) [a]	.04	-.52**	-.09	.09	.13*	-.00	-.04	.00	.36**	.64**	.81**	-								
13. Personal norms (lasagna) [a]	-.04	-.27**	.08	.04	.21**	.11*	.01	.03	.74**	.71**	.57**	.57**	-							
14. Expected satiety (lasagna) [a]	-.02	-.24**	.14*	.08	.20**	.15**	.07	.08	.74**	.63**	.51**	.47**	.81**	-						
15. Portion size selection (spaghetti) [a]	-.11*	-.29**	.16**	.08	.20**	.14*	.26**	.06	.68**	.43**	.38**	.30**	.60**	.57**	-					
16. Perceptions of portion size normality (spaghetti) [a]	.02	-.28**	-.04	.09	.11*	.01	.01	.05	.42**	.62**	.51**	.50**	.56**	.49**	.61**	-				
17. Perceptions of descriptive social norms(spaghetti) [a]	-.00	-.56**	-.08	.06	.10	.02	.05	.01	.34**	.49**	.79**	.71**	.44**	.38**	.46**	.66**	-			
18. Perceptions of injunctive social norms (spaghetti) [a]	.01	-.51**	-.09	.12*	.12*	-.02	.02	-.04	.29**	.50**	.69**	.73**	.47**	.37**	.43**	.63**	.86**	-		
19. Personal norms (spaghetti) [a]	-.09	-.31**	.05	.09	.17**	.01	.05	.02	.55**	.58**	.50**	.48**	.72**	.61**	.78**	.75**	.61**	.60**	-	
20. Expected satiety (spaghetti) [a]	-.09	-.31**	.15**	.11	.20**	.03	.07	.08	.55**	.50**	.45**	.41**	.64**	.72**	.78**	.66**	.55**	.50**	.81**	-
Mean (or number)	38.38	213	26.61	04:40	4.36	7.09	6.97	307	3.95	3.71	3.95	3.50	3.60	4.19	3.74	3.51	3.78	3.40	3.45	3.87
SD (or %)	12.03	65.1%	5.84	04:10	2.30	2.10	2.05	93.3%	2.25	1.62	1.91	1.69	1.78	1.91	1.79	1.35	1.64	1.49	1.49	1.63

* $p < 0.05$. ** $p < 0.01$. [a] Measured by a 9-point scale (range 1-9). [b] Spearman correlation. [c] n unless otherwise stated. Note: All reported means and standard deviations are untransformed scores for ease of interpretation.

Table S3. Portion size selection and portion size evaluations per condition on day 2 in the subsample of participants who correctly recalled the manipulation they were exposed to (n = 204, Study 1).

	**Smaller portion size condition (n = 67)**	**Larger portion size condition (n = 33)**	**Control condition (n = 104)**			
	Mean (SD)	Mean (SD)	Mean (SD)	Test statistic	p-value	η_p^2
Effect of condition on portion size selection						
Portion size selection [a]	3.60 (2.20)	4.55 (2.35)	3.84 (2.11)	$F(2, 201) = 1.74$	0.18	0.02
Effect of condition on perceptions of portion size normality						
Perceptions of portion size normality [a]	3.28 (1.49)	4.45 (1.44)	3.71 (1.55)	$F(2, 201) = 6.63$	0.002	0.06
Effect of condition on perceptions of descriptive norms, injunctive norms, personal norms and expected satiety						
Perceptions of descriptive norms [a]	3.46 (1.90)	4.65 (1.69)	3.94 (1.95)	$F(2, 201) = 5.08$	0.01	0.05
Perceptions of injunctive norms [a]	3.01 (1.56)	4.27 (1.70)	3.57 (1.76)	$F(2, 201) = 5.88$	0.003	0.06
Personal norms [a]	3.33 (1.91)	4.17 (1.50)	3.44 (1.71)	$F(2, 201) = 3.72$	0.03	0.04
Expected satiety [a]	3.77 (1.89)	4.76 (1.60)	4.06 (1.91)	$F(2, 201) = 3.77$	0.03	0.04

[a] Measured by a 9-point scale (range 1-9). Note: All reported means and standard deviations are untransformed scores for ease of interpretation.

Table S4. Component paths of the indirect effect of condition on consumption day 2 for each proposed mediator (*n* = 132, Study 2).

	**Relation between condition and proposed mediator** *B* (95% CI)	**Relation between proposed mediator and consumption** *B* (95% CI)
Perceptions of portion size normality	0.08 (-0.05, 0.21)	33.44 (17.96, 48.91)
Perceptions of descriptive social norms	0.18 (0.05, 0.32)	42.23 (24.28, 60.18)
Perceptions of injunctive social norms	0.16 (0.04, 0.28)	37.77 (18.94, 56.60)
Personal norms	0.09 (-0.06, 0.24)	40.59 (27.01, 54.18)
Expected satiety	0.09 (-0.05, 0.23)	28.59 (15.51, 41.67)

B = unstandardized regression coefficient. CI = confidence interval.

Table S5. Pearson's correlations between main variables (*n* = 132, Study 2).

Variable	1	2	3	4	5	6	7	8	9	10	11	12	13
1. Age	-												
2. BMI (kg/m^2)	.07	-											
3. Baseline hunger (session two) [a]	.08	.02	-										
4. Liking (session two) [a]	-.17*	-.09	.16	-									
5. Frequency of eating lasagna [a]	.11	-.04	.08	-.05	-								
6. Awareness of monitoring consumption [a]	.13	.04	-.03	-.02	.12	-							
7. Nationality (Dutch) [b]	.12	.00	-.09	-.04	.08	-.09	-						
8. Consumption (grams)	.18*	-.04	.28**	.20*	.24**	.09	-.01	-					
9. Perceptions of portion size normality [a]	.02	-.12	.04	.13	.05	.12	-.06	.35**	-				
10. Perceptions of descriptive social norms [a]	-.01	.02	.12	.17*	-.01	.07	.03	.38**	.73**	-			
11. Perceptions of injunctive social norms [a]	.04	-.04	.09	.04	.03	.09	-.07	.33**	.70**	.70**	-		
12. Personal norms [a]	.00	-.13	.14	.17	.07	.17*	-.04	.46**	.82**	.71**	.75**	-	
13. Expected satiety [a]	-.10	-.04	.15	.11	.04	.14	-.05	.36**	.68**	.62**	.66**	.81**	-
Mean (or number)	20.92	21.87	7.05	6.08	5.17	6.92	125	425.84	3.20	2.82	2.92	3.18	3.80
SD (or %)	2.03	2.31	1.51	1.52	.95	2.11	94.7%	121.00	1.27	1.08	1.05	1.37	1.50

* $p < 0.05$. ** $p < 0.01$. [a] Measured by a 9-point scale (range 1-9). [b] Spearman correlation. Note: All reported means and standard deviations are untransformed scores for ease of interpretation.

Table S6. Portion size selection, consumption and portion size evaluations per condition on day 2 in the subsample of participants who correctly recalled the manipulation they were exposed to (n = 92, Study 2).

	**Smaller portion size condition (n = 68)**	**Larger portion size condition (n = 24)**			
	Mean (SD)	Mean (SD)	Test statistic	p-value	d
Effect of condition on consumption					
Portion size selection (grams)	401.64 (115.25)	520.10 (173.19)	$t(90) = -3.77$	< 0.001	0.89
Consumption (grams)	382.57 (104.70)	468.28 (139.47)	$t(90) = -3.15$	< 0.01	0.75
Effect of condition on perceptions of portion size normality					
Perceptions of portion size normality [a]	3.04 (1.09)	4.04 (1.60)	$t(90) = -3.07$	< 0.01	0.81
Effect of condition on perceptions of descriptive norms, injunctive norms, personal norms and expected satiety					
Perceptions of descriptive norms [a]	2.59 (1.03)	3.58 (1.09)	$t(90) = -3.93$	< 0.001	0.94
Perceptions of injunctive norms [a]	2.68 (0.91)	3.90 (1.17)	$t(90) = -4.85$	< 0.001	1.24
Personal norms [a]	2.99 (1.14)	4.15 (1.68)	$t(90) = -3.21$	< 0.01	0.89
Expected satiety [a]	3.60 (1.36)	4.77 (1.43)	$t(90) = -3.43$	< 0.01	0.85

[a] Measured by a 9-point scale (range 1-9). Note: All reported means and standard deviations are untransformed scores for ease of interpretation.

Table S7. Additional consumption results per condition (n = 132, Study 2).

	**Smaller portion size condition (n = 68)** [b]	**Larger portion size condition (n = 64)** [c]			
	Mean (SD)	Mean (SD)	Test statistic	p-value	d
Salad consumption day 1 (grams)	9.24 (2.05)	8.44 (2.71)	t(117.34) = 1.90 [a]	0.06	0.33
Salad consumption day 2 (grams)	15.96 (8.58)	16.24 (8.01)	t(127) = -0.19	0.85	0.03

[a] Results of a Welch's t-test. [b] n = 67 for salad consumption day 2 (because of unusual observations). [c] n = 62 for salad consumption day 2 (because of missing and usual observations).
